# Senescence of the immune defences and reproductive trade-offs in females of the mealworm beetle, *Tenebrio molitor*

**DOI:** 10.1038/s41598-022-24334-y

**Published:** 2022-11-17

**Authors:** Charly Jehan, Camille Sabarly, Thierry Rigaud, Yannick Moret

**Affiliations:** Laboratoire BioGéoSciences, UMR CNRS 6282, Équipe Écologie Évolutive, Université Bourgogne-Franche Comté, 6 Boulevard Gabriel, 21000 Dijon, France

**Keywords:** Ecology, Evolutionary ecology

## Abstract

In the theory of ageing, it has been assumed that ageing is associated with a decline in somatic defences, including the immune system, as a consequence of a trade-off with reproduction. While overall immunity suffers from age-related deterioration (immune senescence), the different components of the immune response appear to age differently. It is also likely that investment among the many arms of the immune system and reproduction with age is finely adjusted to the organisms' reproductive strategy. We investigated this possibility in females of *Tenebrio molitor*, a species of long-lived insect with reproductive strategies similar to those of long-lived mammals. We specifically tested the effects of immunological challenges imposed early or late in adult life on immune pathway activation as well as fertility early and late in life. We found complex patterns of changes in immune defences with age and age-specific immune challenges with contrasted relationships with female reproduction. While cellular and enzymatic defences showed signs of ageing, they did not trade-off with reproduction. By contrast, the induced antibacterial immune response was found to be unaffected by age and to be highly connected to female fecundity. These findings suggest that these immunological pathways have different functions with regard to female ageing in this insect species.

## Introduction

Central to the evolution of life history is the principle of resource allocation^1^, which posits that as an individual’s resources are limited, they should be optimally allocated between growth, reproduction, and survival to maximize fitness^2^. The disposable soma theory of ageing, which predicts that reproduction trades-offs with somatic protection and maintenance, leading to a progressive decline in future reproduction, disease resistance, and survival, is based on this premise^3^.

While the relationship between reproduction allocation and senescence is starting to be well documented in some vertebrates and invertebrates from both natural and laboratory populations^4–8^ the determinants of this relationship are still poorly studied. Among them, immune defence is predicted to play a central role. The immune system strongly contributes to the protection of the soma and has a critical role in evolutionary fitness as it protects its host from infection by parasites and pathogens^9^. Ageing is often accompanied by the diminished capacity of the immune system, known as immunosenescence, which results in increased sensitivity to infections with age. Immune defence and reproduction are demanding functions, both physiologically and energetically, and have been observed to trade-off in a diversity of organisms^10^. Increased reproductive effort can result in reduced immunity, and reciprocally, infection and activation of the immune system can reduce reproductive output. However, little is known about the implications of such a relationship on age-related declines of physiological functions and individual survival probability. Given that the cost of reproduction is thought to decrease somatic defences^1,3,7,11^, it is reasonable to predict that more reproductive effort would lead to stronger immunosenescence. However, depending on the species’ evolved reproductive tactics to maximize lifetime reproductive success, the detection and intensity of the cost of reproduction vary. Accordingly, in long-lived species like large mammals, the cost of current reproduction is known to reduce future reproduction rather than longevity, whereas the opposite is observed in short-lived animals like rodents^12^. Therefore, compared to short-lived species, long-lived organisms might have developed strategies that avoid jeopardizing immunity at older ages.

Apart from the energetic cost of immunity, increased immune system activity may also contribute to senescence through immunopathology caused by an over-reactive inflammatory immune response to infection^13–16^. Inflammation is an important reaction to injury and infection because it is a key process in pathogen resistance and tissue repair^17^. However, the initial acute inflammatory response may have negative long-lasting health implications if damaged tissues are not fully repaired and homeostasis is not fully restored, resulting in increased rates of morbidity and mortality at older age^18–20^. Accordingly, an early inflammatory immune response can be associated with a shorter lifespan in an insect^21,22^. However, little is known about the long-term subsequent consequences of the immune response on reproductive and immune senescence. Furthermore, like the cost of reproduction, the subsequent impact of the immune response on reproductive and immune senescence is likely to vary depending on the organism’s reproductive tactic. Therefore, an early immune response may cause stronger immunosenescence in short-lived organisms than in long-lived organisms.

We tested the effects of age-specific immune challenges on early and late-life fecundity and immune activity in females of the mealworm beetle, *Tenebrio molitor*. The mealworm beetle is a pest of stored grain products that is gaining interest as an alternative protein source for food and feed^23^. Females can begin reproducing as early as the fifth day after eclosion and mate multiple times with several males during their adult life of 12 to 20 weeks^24^. This insect is a relatively long-lived insect species with reproductive tactics similar to those of long-lived mammals^8^, in which the cost of current reproduction compromises future reproduction without jeopardizing survival^12^. Furthermore, females exposed to an immune challenge mimicking a microbial infection had their reproductive output reduced without affecting their longevity^25^. While this result is consistent with *T. molitor* females’ reproductive strategy, it also suggests that the cost of the immune response affects reproduction but has a limited impact on somatic defences later in life. As a result, we hypothesized that mounting an immune response early in life may have a limited effect on female beetle immunosenescence.

In *T. molitor*, the immune response relies on constitutive and inducible innate mechanisms with cellular and humoral components^23^. Cellular immunity is mediated by four types of haemocytes: prohemocytes, granulocytes, plasmatocytes, and oenocytoids, which are responsible for phagocytosis, nodulation, and encapsulation of endogenous organisms^23,26–28^. Humoral effectors are efficient parts of the innate immune response, including but not limited to the activation of the prophenoloxidase (proPO) system, whose enzymes are constitutively synthesized and located both in the haemolymph and granulocytes^28^. The activation of the proPO enzyme is at the core of the inflammatory response^29,30^, as it produces cytotoxic compounds^31^, causing self-damage and eventually life span reduction^21,22,32–34^. In addition, microbial infections also stimulate the synthesis and secretion of antimicrobial peptides into the haemolymph^35–40^. They may last several days, either to mop up any microbial pathogens escaping immune cells and phenoloxidase activity^41^ or to prepare the insect for subsequent pathogenic attacks^42^. Antimicrobial peptides are relatively specific and kill microbes without causing harm to the host’s tissues^43^. At older age, this immune pathway may therefore take precedence over cellular defence and the prophenoloxidase system^44^.

**Figure 1:**
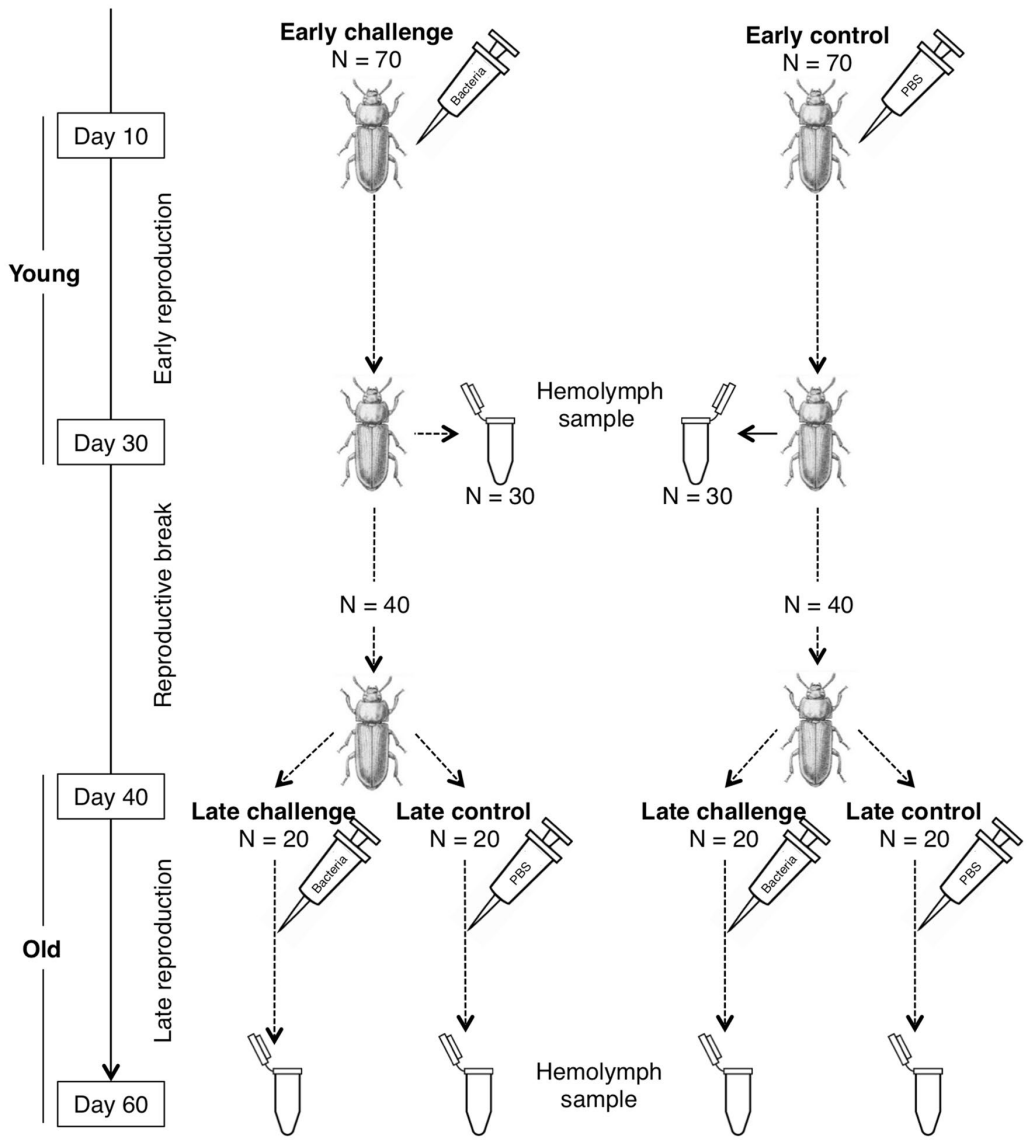
The experimental protocol used to study the effect of an age-specific immune challenge on the immune defences and reproduction of females of the mealworm beetle (*Tenebrio molitor*).

In this study, age-controlled females of *T. molitor* were immune challenged early (10 days post eclosion) or late in life (40 days post eclosion) with an injection of inactivated *Bacillus cereus* to mimic a systemic bacterial infection, inducing an inflammatory immune response and increasing investment in somatic defences (Fig. 1). The effect of these age-specific immune challenges on female subsequent immunity and fecundity was investigated (Fig. 1). Immunity was determined by measuring the concentration of the four different types of haemocytes, prophenoloxidase enzyme activity, and antibacterial activity at the end of the early or late-life reproductive episode (Fig. 1). We found complex patterns of changes in immunity with age and the age at which the immune challenges were performed, with contrasting relationships with female reproduction. While cellular and enzymatic defences showed signs of senescence, there was no direct link to reproduction. By contrast, the induced antibacterial immune response was found to be unaffected by age but strongly linked to female reproduction. These results suggest that these immune pathways serve different functions in relation to the life history of females in this insect species.

## Results

### Fecundity declines with age and as a result of an early immune challenge in young females

**Figure 2:**
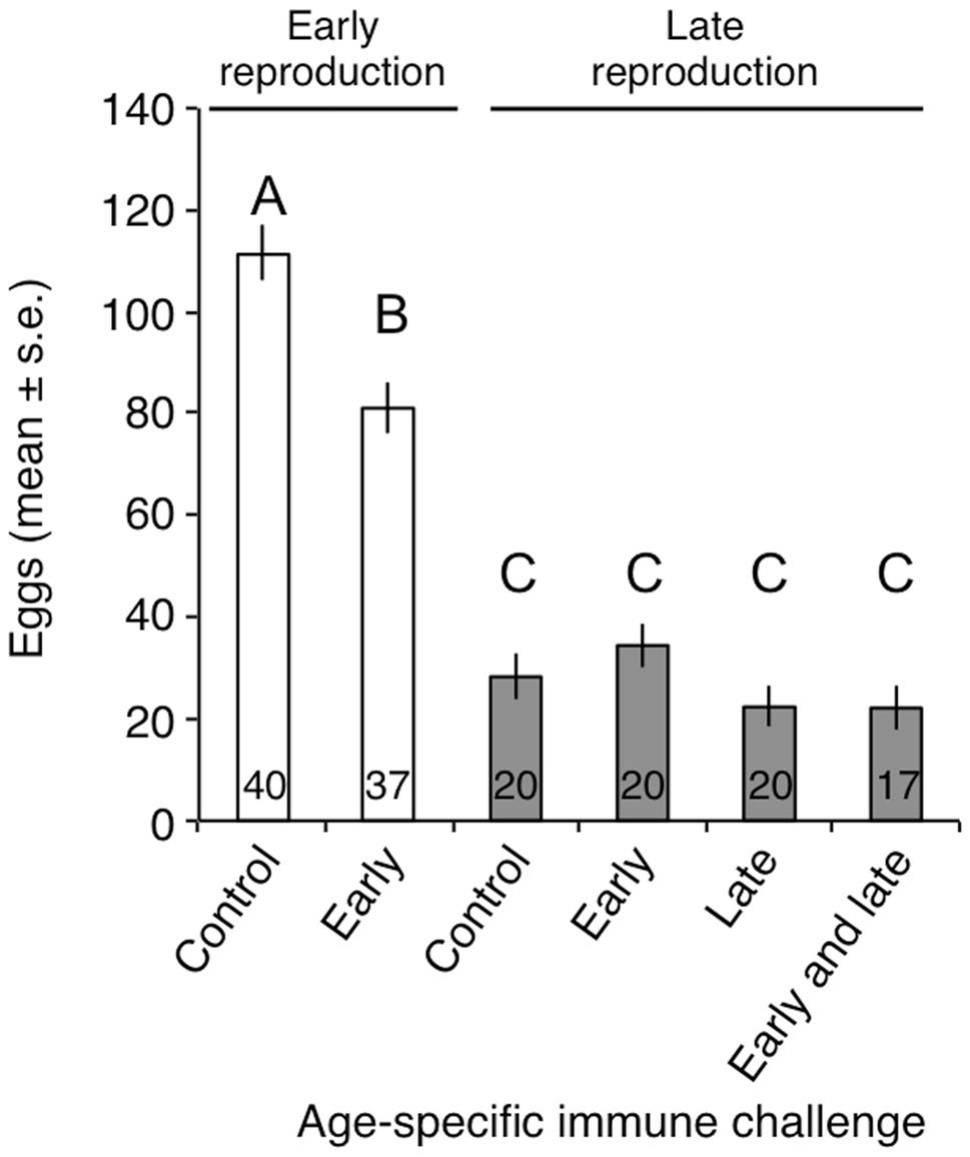
Mean number of eggs (± s.e.) produced by females early in life (young: between day 10 and day 30 post eclosion) and late in life (old: between day 40 and day 60 post eclosion) as function of their life time immune treatment: control, immune challenged early only (early), immune challenged late only (late) and immune challenged both early and late in life (early and late). The numbers at the bottom of the bars are the sample size (the number of females involved). Bars topped with the same capital letters denoted groups with non-significant differences (see Table 1).

As previously observed in another study^25^, female fecundity decreased with age and because of the early immunological challenge, both as main effects and as a two-way interaction term (Table 1). According to the early immune challenge and age interaction term, the early immunological challenge only resulted in a reduction in female fecundity at a young age (Fig. 2). Whether or not the females had previously been subjected to an early challenge, the late immunological challenge had no discernible impact on their fecundity (Table 1 and Fig. 2). Female body mass explained a significant portion of the variation in female fecundity, with the heaviest females laying more eggs than the lightest (Table 1).

**Table 1:** Mixed linear model investigating variance in female egg production as a function of their age, their experience of an immune challenge occurring early or late in life and body mass measured before each reproductive episode. Female identity was included as a random factor. Values with *P* ≤ 0.05 are given in bold. This analysis was done on data from females that reproduced both early and late in life.

Source	Estimate ± s.e	d.f. _num_	d.f. _den_	F	*P*
Age^a^	53.70 ± 7.22	1	155	159.02	** < 0.001**
Early challenge^b^	−1.15 ± 6.66	1	155	6.25	**0.013**
Late challenge^b^	1.63 ± 4.72	1	155	0.12	0.730
Body mass	0.25 ± 0.12	1	155	4.15	**0.043**
Age *Early challenge	25.84 ± 9.38	1	155	7.59	**0.007**

^a^Comparison of young females relative to old females, which are used as the reference category (estimate = 0).

^b^Comparison of control females relative to challenged females, which are used as the reference category (estimate = 0).

### Immune parameters

No oenocytoids and very few prohaemocytes were found in the haemolymph samples of the females. Prohaemocytes were observed in the haemolymph of 4 young females (30 days post-eclosion) only. Therefore, data on prohaemocytes could not be used for further statistical analyses.

Using a Principal Component Analysis (PCA) on the immunological measures (e.g., granulocytes, plasmatocytes, total haemocytes, PO, total PO, and antibacterial activity) a large proportion (85.44%) of the variance could be summarized into 3 main components (PC1: 41.85%, PC2: 26.93%, and PC3: 16.67%). Cellular immune defences: total haemocytes, granulocytes, and plasmatocytes, mostly contributed to the first axis (PC1, factor loading for total haemocytes: 0.98, granulocytes: 0.96, and plasmatocytes: 0.77). Enzymatic defences mostly contributed to the second axis (PC2, factor loading for PO activity: 0.90, and total-PO activity: 0.89). The inducible antibacterial activity mostly contributed to the third axis (PC3, factor loading for antibacterial activity: 0.99). The contribution of each immunological measure on all the three axes is detailed in supplementary materials (Table S1).

**Figure 3:**
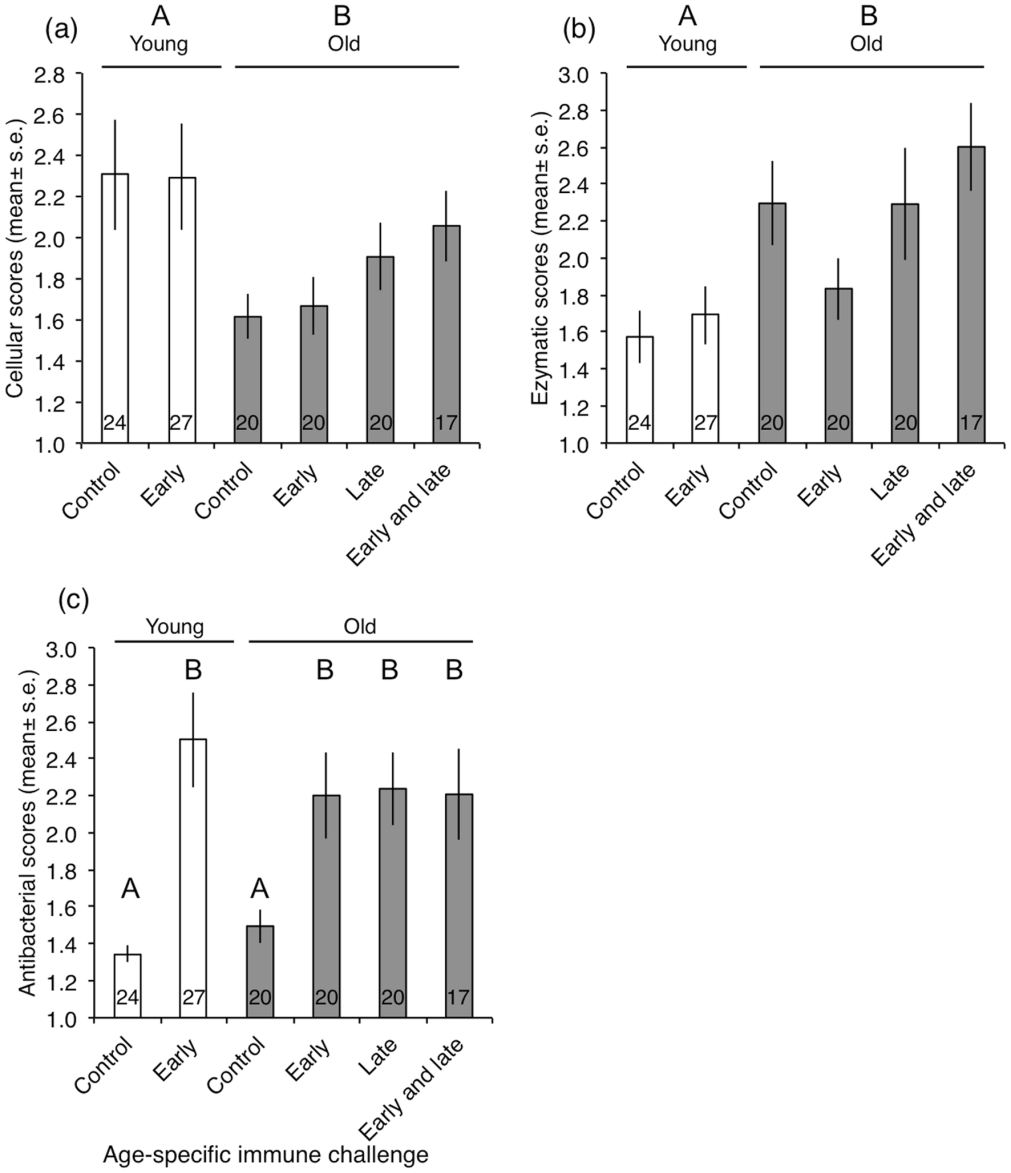
Summarized immune scores for (**a**) cellular, (**b**) enzymatic, and (**c**) antibacterial defences according to the age at which females were sampled for their haemolymph (young: at 30 days post-eclosion and old: at 60 days post-eclosion) and as a function of their age-specific immune treatment (early control and early challenge at 10 days post-eclosion, and late control and late challenge: at 40 days post-eclosion). The numbers at the bottom of the bars are the sample size (the number of females involved). Bars or groups topped with the same capital letters denoted groups with non-significant differences (see Table 2).

The cellular and enzymatic immune scores (PC1 and PC2, respectively) were only explained by the age at which females were sampled for their haemolymph (Table 2a,b). Older females had lower cellular immunity (Fig. 3a) but higher levels of enzymatic immunity (Fig. 3b) than younger ones. The removal of the late-life immune challenge did not improve the statistical models for both the cellular and enzymatic immune scores (Table 2a,b), indicating that this factor may contribute to the variation of these immune scores, though its effect was not strong (*P* > 0.05). Indeed, the late-life immune challenge tends to increase both cellular and enzymatic immune scores (Fig. 3a,b).

**Table 2:** General Linearized Models (GLMs) for the summary immune values obtained from the principal component analysis (cellular immunity as PC1; enzymatic immunity as PC2 and inducible immunity as PC3) as a function of age of haemolymph sample (40 *versus* 60 days post emergence), their early-life immune treatment (control *versus* early challenge), their late-life immune treatment (control *versus* late challenge), body mass measured during haemolymph sampling and their lifetime fecundity. The best models were searched from a full model involving the above covariates as main effects and two-way interaction terms. Body mass was included as a main effect only. Model simplification was achieved by removing non-significant covariates based on Akaike’s information criterion (ACI). Simplification stopped when the reduced model did not contribute to a reduction of at least 2 AIC units compared to the previous model. The Omnibus test assesses whether the model significantly explains variation against the null model.

Source of variation		ß	s.e	Num. d.f	Denom. d.f	χ^2^	*P*
**(a) Cellular immunity (PC1)**^**a**^							
Omnibus test				2	125	12.32	**0.002**
Age^b^		0.34	0.10	1	125	12.50	** < 0.001**
Late challenge^c^		−0.19	0.10	1	125	3.23	0.072
**(b) Enzymatic immunity (PC2)**^**a**^							
Omnibus test				2	125	16.11	** < 0.001**
Age^b^		−0.23	0.10	1	125	5.62	**0.018**
Late challenge^c^		−0.16	0.11	1	125	2.38	0.123
**(c) Inducible immunity (PC3)**^**a**^							
Omnibus test				5	122	66.77	** < 0.001**
Early challenge ^c^		−0.35	0.21	1	122	11.07	**0.001**
Late challenge ^c^		−0.02	0.11	1	122	11.41	**0.001**
Fecundity		−0.004	0.001	1	122	12.34	** < 0.001**
Early challenge*Late challenge		−0.45	0.15	1	122	9.38	**0.002**
Early challenge*Fecundity		0.003	0.001	1	122	6.37	**0.012**

^a^(value + 2): ‘gamma’ distribution with a Log link function.

^b^Comparison of old female relative to young females, which are used as the reference category (estimate = 0).

^c^Comparison of challenged female relative to controls, which are used as the reference category (estimate = 0).

Variation in the inducible antibacterial immune score (PC3) is explained by the interaction between the early-life immune challenge and the late-life challenge (Table 2c). While an immune challenge, whether early or late in life, resulted in an enhanced antibacterial immune score, the late-life immune challenge did not induce an additional increase in antibacterial activity in females who had previously been immune challenged (Fig. 3c).

**Figure 4:**
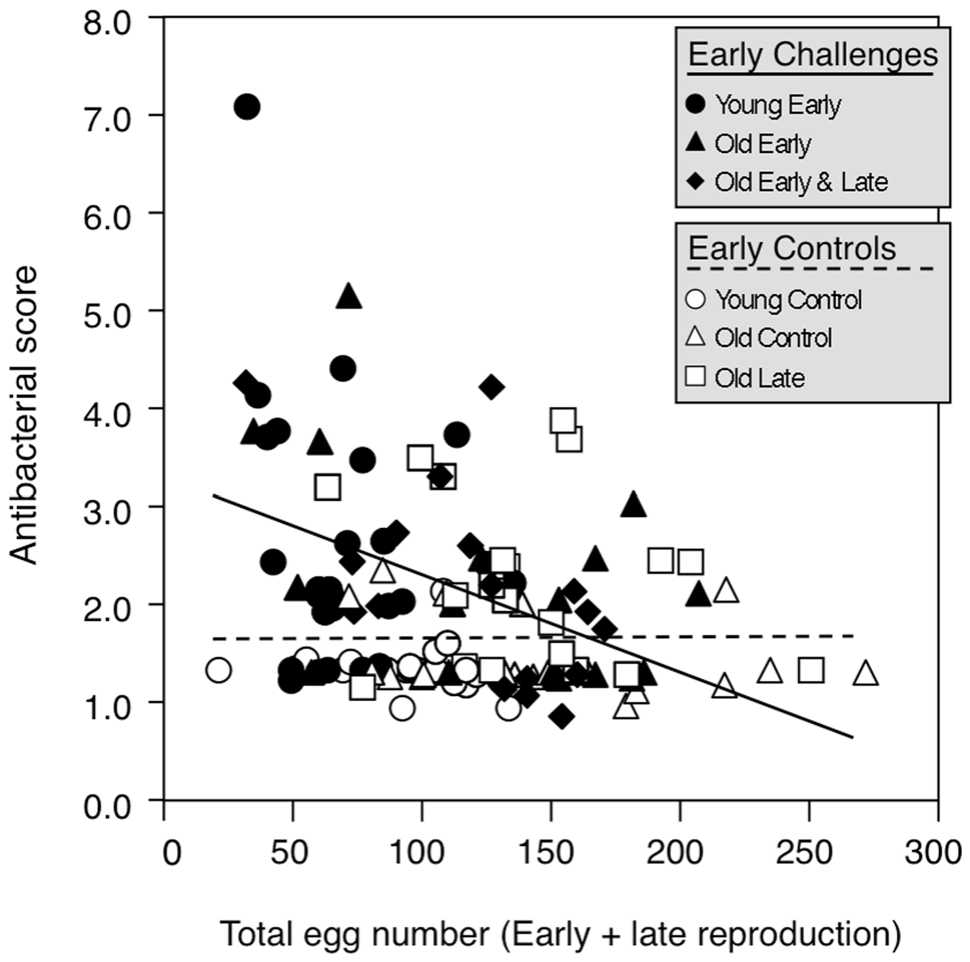
Variation in scores of inducible antibacterial defence as a function of total fecundity (number of eggs laid) the early-life immune treatment. The negative relationship between antibacterial scores and fecundity is significant only among females that were exposed to an early immune challenge (continuous line: F_1, 62_ = 11.77, *P* = 0.001, r = 0.4). Among controls, the relationship is not statistically significant, and the dashed line is illustrative only.

Furthermore, the antibacterial immune score was found dependent on the interaction between the early immune challenge and female fecundity (Table 2c). The negative correlation between fecundity and antibacterial immune score in females with antibacterial activity explains this result (Fig. 4). Since antibacterial activity is inducible, an early immune challenge is required to reveal the relationship, especially among young females. As females age, the probability of being naturally exposed to a bacterial infection increases, resulting in a higher prevalence of antibacterial activity among control old females than among control young ones (Fig. 3c), explaining the absence of an interaction between late-life immune challenge and fecundity (Table 2c).

## Discussion

Breeding females of *T. molitor* exhibited measurable variation in immune defences according to age, age-specific immune challenges and fecundity. However, the different components of the immune system showed different patterns of variation according to these factors. Indeed, among the three main immune defence components, only the cellular and the enzymatic defences were found to co-vary with age. While cellular defences were found to decrease with age, enzymatic ones were found to increase. These results confirm previous observations of age-specific variation in cellular and enzymatic defences in *T. molitor*^8,24,45^ and in other insects^46–50^.

Previous evidence suggests that females of the mealworm beetle exhibit comparable life history tactics to those of long-lived mammals in that the cost of reproduction is reflected in future reproduction rather than survival^8,12,25^. Furthermore, an immunological challenge simulates an increased likelihood of impending infection-related death. However, it has been demonstrated that immunological challenges administered to *T. molitor* females either early or late in life do not cause them to invest their remaining energy in procreation at the price of their longevity^25^. By contrast, females appear to reduce the cost of current reproduction by restraining it, possibly to gain additional subsequent reproductive opportunities later in life^8,25^. Such a life history tactic implies that females should sustain a certain level of investment in somatic defences, including immunity, at old age. The results of our current study indeed confirm this possibility by showing that antibacterial activity and, to a lesser extent, cellular and enzymatic defences increase in response to an immune challenge late in life.

The immune challenges were found to have no or very weak effects on cellular defences. At best, only the late immune challenge marginally increased the concentration of immune cells in the haemolymph of females. The concentration of immune cells usually increases rapidly following an immune insult and then goes back to ground level after a few days^51,52^. Here, the relatively long lag time of 20 days between the immune challenge and haemolymph sampling may explain such a weak effect.

The increased activity of enzymatic defences with age may appear surprising in the context of ageing, especially since it has been previously shown that old individuals are less resistant to bacterial infections than young ones^8^ and that old males are less able to melanise a nylon monofilament implanted in the body cavity than young ones^33^. The prophenoloxidase system is perhaps the most important constitutive immune effector system in insects^53^. The proPO activation cascade is regulated by plasma serine protease inhibitors and active phenoloxidase might be directly inhibited by proteinaceous factors^54^. Such a regulation is essential because the products of PO activity are potentially toxic to the host ^21,22,55^. The observed increased activity of the proPO cascade late in life could therefore be in line with a progressive dysfunction of such a regulation system with age. Furthermore, the age-related decline of immune cells, which contain the enzymatic complex of the proPO system, may explain the previously observed reduced encapsulation response of implanted nylon monofilament by old males^33^. Under this hypothesis of an age-related increased PO activity dysregulation, older females may fail to restore homeostasis and suffer from an enhanced chronic inflammatory status^22^, which could explain their lower resistance to infection^8^ and their increased late-life mortality risk^22^. The relationship between PO activity dysregulation and late-life survival probability will need to be investigated further.

The induced antibacterial activity was unrelated to female age and, once triggered early in life, it remained consistent and high late in life, regardless of whether the females were exposed to another challenge or not. This suggests that antibacterial peptide synthesis is less susceptible to senescence than cellular and enzymatic defences. In contrast to cellular immunity and the proPO system, antibacterial peptides do not damage host tissues^43^. Furthermore, relative to molecular size and assuming an equivalent efficiency against parasites, the inducible synthesis of antibacterial peptides might be less costly per unit than the constitutive production of immune cells and enzymes of the proPO system^29,43^, making this immune pathway provide the cheapest immunity^44^. While it has the disadvantage of a relatively long lag-phase during synthesis in response to infection, its efficacy relies on the long duration of the immune response, suggesting its relatively low cost. Here, we confirm this long duration with the maintenance of high antimicrobial activity for at least 50 days post challenge.

Patterns of changes in immune defences with age and as a consequence of age-specific immune challenges are therefore complex according to the immune parameters considered, possibly driven by their respective costs. Such a complexity remains when considering female reproduction. Reproduction has previously been shown to affect cellular components of *T. molitor*'s immune system^8^. In this previous study, young and old females were allowed or not to reproduce, and those allowed to reproduce either early, late, or both early and late in life exhibited different haemocyte counts than those that did not reproduce (higher or lower counts, according to the haemocyte type considered). Here, all the females were allowed to reproduce, and cellular immune scores did not correlate with fecundity. While this suggests the absence of a direct trade-off between cellular immunity and reproductive effort, it also implies that the previously observed changes in cellular immune defences induced by reproduction^8^ are primarily caused by injuries during copulations, as found in other insects^56^.

Enzymatic defences and female fecundity were not found correlated. Previous evidence showed that cuticle melanisation in *T. molitor*, which indicates higher constitutive phenoloxidase activity^57^, did not correlate with fecundity^58^. Under the hypothesis that reproduction comes at a cost in terms of PO activity dysregulation, it may be worthwhile to investigate the relationship between reproduction and the expression or the activity of the proteins involved in the proPO system regulation^54^.

After an early immune challenge, induced antibacterial activity was found to be unrelated to age, but highly negatively correlated with fecundity. This is the first evidence of a direct trade-off between reproductive effort and the production of immune effectors at the individual level. This trade-off suggests that both functions compete for the same pool of resources stored in shared tissues. The fat body of insects has been proposed to be the control centre for the reproduction-immunity trade-off^10^. On the one hand, this organ is critically important for oogenesis because it is the major site for yolk protein and vitellogenin production for oocytes^57,58^. On the other hand, it is also a primary organ of systemic immunity^61^. In particular, the production of antimicrobial peptides in response to a microbial challenge is mostly located in the cells of the fat body before being secreted into the haemolymph^62^. Finding a direct negative relationship between antimicrobial activity and fecundity in challenged females makes sense.

While reproductive effort appears to impose unequal trade-offs with the different immune parameters assessed in our study, it is noticeable that females exposed to the early immune challenge exhibited reduced early fecundity. Assuming that reproduction and immunity are mutually limiting, this implies that the cost of long-term antibacterial peptide production is significant early in life, while the cost of maintenance is relatively modest later. Indeed, the synthesis of antibacterial peptides in response to the early immune challenge may have lowered early fecundity, but once it has been triggered, the reproductive cost late in life may remain relatively low. By contrast, cellular and enzymatic defences tend to have lower upfront developmental costs but higher operating costs with a late-life impact that could shorten lifespan^21,22^. Cellular and enzymatic defences are mobilized quickly and effectively against novel infections^63^, whereas the antibacterial immune response is less effective against novel exposures to microbial pathogens, but more effective once activated against secondary exposures due to a priming effect^42,64^. Such a priming response, maintaining high antibacterial activity late in life, is consistent with the apparent restrained reproductive tactic used by older females to improve survival against pathogenic threats late in life and benefit from future reproductive opportunities^8,25^. Therefore, patterns of age-specific changes in the mealworm beetle’s immunological pathways might be linked to their respective functions based on the insect’s life history.

## Methods

### Insect culture and experimental design

Virgin adult beetles of controlled age (10 ± 2 days post-eclosion) were obtained from pupae, randomly sampled from a stock culture, and maintained in standard laboratory conditions (24 ± 1 °C, 70% RH in permanent darkness). Prior to the experiment, all the experimental insects were kept separately in standard laboratory conditions (see above), fed ad libitum bran flour and water, and given a piece of apple once a week.

We assessed the effects of age-specific immune challenges on reproductive performance and immunity of *T. molitor* females exposed to early or late-life immune challenges (Fig. 1). For this purpose, a group of seventy 10-day-old (10-day post-eclosion) virgin females were individually weighed to the nearest 1 mg using an OHAUS balance (discovery series, DU114C). They were then immune challenged with a single 5-µL injection of an inactivated *Bacillus cereus* suspension (10^8^ bacteria.mL^−1^ in Phosphate Saline Buffer: PBS 10 mM, pH 7.4) to mimic a systemic bacterial infection (see below for the inactivation procedure). *Bacillus cereus* is a natural pathogen of *T. molitor* ^65,66^ known to stimulate its immune response and to induce significant mortality upon infection^67^. As a procedural control, another group of 70 females received a 5-µL injection of sterile PBS. In adults *T. molitor*, injection of sterile solution may induce a low and short-lived antibacterial immune response^63^, but previous work showed it had no effect on reproduction^25,68^ or survival compared to naïve beetles^21,22^. *T. molitor* wounds frequently, and the injection of sterile saline solution appears insignificant in this context. For this reason, the experiment did not include a group of unmanipulated females. Females were allowed to reproduce for 20 days (from day 10 to day 30 post-eclosion, Fig. 1) immediately following their respective immune treatments to assess their early-life reproductive performance, as described in^8,25^. In brief, each female was paired with a 10 ± 2-day-old virgin male for 10 days. Then, the male was replaced by a new 10 ± 2-day-old virgin male, with whom the female spent the next 10 days. Couples were maintained in a Petri dish containing bleached flour and provided ad libitum food and water under standard laboratory conditions (24 ± 1 °C, 70% RH in permanent darkness). Female reproductive output was estimated by counting the number of eggs produced by the female every 3 or 4 days during the 20-day period of reproduction. To do this, couples were transferred into a new Petri dish, and the eggs were searched by sieving the flour (600 µm) and counting^68^. Females were 30 days old at the end of this early reproductive period. Thirty of these young females randomly taken from each immune treatment group were individually weighed, and a sample of haemolymph was collected from each to assess their immune activity following early reproduction while being exposed or not to an early-life immune challenge (Fig. 1). Females were sacrificed after haemolymph sampling.

The remaining females from each immune treatment group (40 early challenged females and 40 control females) were kept alone in a new Petri dish for 10 days (until day 40 post-eclosion, Fig. 1), awaiting to be exposed to their late-life immune treatment and allowed to reproduce during a 20-day late reproduction period (from day 40 to day 60 post-eclosion). Within each early-life immune treatment group of females, one half was individually immune challenged with a 5-µL injection of the inactivated *B. cereus* suspension, while the other half received a 5-µL injection of PBS as a control (Fig. 1). This enabled us to generate 4 groups of 20 females who were immune challenged early in life only, late in life only, both early and late in life, and never challenged (control). Females were then allowed to reproduce a second time, as previously described. At the end of this late reproductive period, each female was weighed and a sample of haemolymph was collected to assess immune activity late in life according to age-specific immune challenge and reproductive effort (Fig. 1). Females were sacrificed after heamolymph sampling.

### Bacterial cultures for immune challenges

Bacterial cultures for immune challenges were done as described by^8^. The bacterium *Bacillus cereus* used in the immune challenges was obtained from the Pasteur Institute (CIP69.12). The bacteria were cultivated overnight at 28 °C in a liquid broth medium (10 g/L bacto-tryptone, 5 g/L yeast extract, 10 g/L NaCl, pH 7). Bacteria were then adjusted to have a concentration of 10^8^ bacteria per mL using a Neubauer improved cell counting chamber after being inactivated in 0.5% formaldehyde prepared in phosphate-buffered saline (PBS 10 mM, pH 7.4) for 30 min. The success of the inactivation was tested by plating a sample of the bacterial solution on sterile broth medium with 1% of bacterial agar and incubating it at 28 °C for 24 h. Aliquots were kept at −20 °C until use. Immunological challenges were administered by injection through the pleural membrane between the second and third abdominal tergites using sterile glass capillaries pulled out to a fine point with an electrode puller (Narashige PC-10).

### Haemolymph collection and immune parameters

Haemolymph collection was done as described by^42^. Briefly, beetles were chilled on ice for 10 min before collecting a first 3-µL sample of haemolymph from a wound in the beetle’s neck. The sample was flushed into a microcentrifuge tube with 15 µL of anticoagulant buffer (30 mM trisodium citrate, 26 mM citric acid, 15 mM sodium chloride, 20 mM EDTA, pH 4.6)^69^. A 10-µL subsample was rapidly used for haemocyte count using a Neubauer improved haemocytometer under a phase-contrast microscope (magnification × 400). The different haemocyte types found in *T. molitor* haemolymph were distinguished using previous description^27^. We especially measured the concentration of granulocytes, plasmatocytes, and prohemocytes. In our study, the observation of oenocytoïds was anecdotic.

Immediately after the first sample of haemolymph, a second 3-µL sample was collected from a new wound in the female neck. This sample was flushed into a microcentrifuge tube with 20 µL of PBS. Five-µL were transferred to an N-phenylthiourea-coated microcentrifuge tube (P7629; Sigma-Aldrich, St Louis, MO, USA), flash-frozen in liquid nitrogen, and stored at −80 °C for later antibacterial activity testing. The remaining haemolymph solution (18 µL) was also flash-frozen in liquid nitrogen and stored at −80 °C until the activity of the prophenoloxidase system was measured^29^.

The activity of the prophenoloxidase system was estimated by (i) the activity of the naturally activated phenoloxidase (PO) enzyme only (hereafter PO activity) and (ii) the activity of PO plus that of the inactivated proenzymes (proPO) (hereafter total-PO activity) using the spectrophotometric assay described in^70^. Total-PO activity quantification was determined by converting proPO enzymes into active PO enzymes with chymotrypsin, whereas PO activity was determined directly from the sample. Frozen haemolymph samples were thawed on ice and centrifuged (3500 g, 5 min, 4 °C). In a 96-well plate, 5 µL of supernatant were diluted in 20 µL of PBS and were added either 140 µL of distilled water to measure PO activity or 140 µL of 0.07 mg. mL-1 chymotrypsin solution (Sigma-Aldrich, St Louis, MO, USA, C-7762) to measure Total-PO activity. Subsequently, 20 µL of a 4 mg.mL-1 L-Dopa solution (Sigma-Aldrich, St Louis, MO, USA, D-9628) were added to each well. The reaction proceeded for 40 min at 30 °C, in a microplate reader (Versamax; Molecular Devices, Sunnyval, CA, USA). Reads were taken every 15 s at 490 nm and analysed using the SOFT-MaxPro software version 4.0 (Molecular Devices, Sunnyvale, CA, USA). Enzymatic activity was measured as the slope (Vmax value: change in absorbance unit per min) of the reaction curve during the linear phase of the reaction and reported as the activity of 1 µL of pure haemolymph.

Antimicrobial activity of the haemolymph was measured using the inhibition zone assay described by^71^. Briefly, haemolymph samples were thawed on ice, and 2 µL of the sample solution was used to measure antibacterial activity on zone inhibition plates seeded with *Arthrobacter globiformis* from the Pasteur Institute (CIP105365). This bacterium was used to check antibacterial activity because it offers the best sensitivity to any antimicrobial activity developed in the mealworm beetle^72^. An overnight culture of the bacterium was added to broth medium containing 1% agar to achieve a final concentration of 10^5^ cells.mL^−1^. Six millilitres of this seeded medium were subsequently poured into each Petri dish. After solidification, sample wells were made using a Pasteur pipette fitted with a ball pump. Two microliters of the sample were deposited in each well. Plates were then incubated overnight at 28 °C and the diameter of each zone of inhibition was measured to the nearest 0.5 mm using a ruler.

### Statistics

Age-specific fecundity of females that reproduced early and late in life was analysed using a mixed linear model, as a function of the age at which females initiated each reproductive period (10 day old *versus* 40 day old), whether the females were exposed to an early immune challenge (yes/no), a late immune challenge (yes/no) and their interaction as fixed categorical factors. Female body mass measured prior to each reproductive period was included as an independent continuous covariate tested as a main effect only.

A principal component analysis (PCA) was performed to obtain synthetic variables for the innate immune parameters, including the different concentrations of haemocytes (total haemocytes, granulocytes, and plasmatocytes), enzymatic activities (PO and total-PO activities), and antibacterial activity. Variation in the resulting summarized scores of these immune parameters was then analysed using General Linearized Models (GLMs) according to age at which females were sampled for their haemolymph (30 or 60 days post-eclosion as a categorical factor), whether the females were exposed to an early-life immune challenge (at 10 days post-eclosion: challenged *versus* control, Fig. 1), a late-life immune challenge (at 40 days post-eclosion: challenged *versus* control, Fig. 1), total lifetime female fecundity (total number of eggs during the early and the late reproductive periods, as a continuous covariate) and female body mass during haemolymph sample (as a continuous covariate). For each immune summary score, the best model was searched from a full model involving the above covariates as main effects and two-way interaction terms. Body mass was included as a main effect only. Model simplification was achieved by removing non-significant covariates based on Akaike’s information criterion (AIC). Simplification stopped when the reduced model did not contribute to a reduction of at least 2 AIC units compared to the previous model. All the analyses used IBM® SPSS® statistics 19 for Macintosh.

## Supplementary Information

Supplementary Information.

## Data Availability

The datasets generated and/or analysed during the current study are available in the Dryad Digital Repository https://datadryad.org/stash/share/Wg0o09Z-ASnjAXAwdg6J_5vHMDg6xaDkak8ykw6Ly2g.
