# Supplementary material for: Senescence of the immune defences and reproductive trade-offs in females of the mealworm beetle, Tenebrio molitor
Source: Sci Rep. 2022 Nov 17;12:19747. doi: 10.1038/s41598-022-24334-y (PMC9671880; doi:10.1038/s41598-022-24334-y)
Supplement: Supplementary file 1 — Supplementary Information. [file 41598_2022_24334_MOESM1_ESM.docx]

**Supplementary Information**

**Table S1**. Contribution of all the measured immune parameters on the three component axes extracted from the Principal Component Analysis (PCA).

|  | Component | | |
| --- | --- | --- | --- |
| Variables | Axe 1 | Axe 2 | Axe 3 |
| Total hemocytes | 0.98 | -0.01 | 0.03 |
| Granulocytes | 0.96 | -0.02 | 0.05 |
| Plasmatocytes | 0.77 | 0.08 | -0.15 |
| PO activity | -0.09 | 0.90 | -0.05 |
| Total-PO activity | 0.13 | 0.89 | -0.01 |
| Antibacterial activity | -0.04 | 0.04 | 0.99 |
